# Hypnosis on patients treated with adjuvant chemotherapy for breast cancer: A feasibility study

**DOI:** 10.1002/cnr2.1732

**Published:** 2022-10-27

**Authors:** Michel Fabbro, William Jacot, Marta Jarlier, Séverine Guiu, Véronique D'Hondt, Stéphane Pouderoux, Patrice Champoiral, Chloé Janiszewski, Isabelle Nicklès

**Affiliations:** ^1^ Medical Oncology Department Montpellier Cancer Institute (ICM), University of Montpellier Montpellier France; ^2^ Institut de Recherche en Cancérologie de Montpellier (IRCM), Inserm U1194, University of Montpellier Montpellier France; ^3^ Biometrics Unit Montpellier Cancer Institute (ICM), University of Montpellier Montpellier France; ^4^ Psycho‐Oncology Unit Montpellier Cancer Institute (ICM), University of Montpellier Montpellier France; ^5^ Clinical Research Center Montpellier Cancer Institute (ICM), University of Montpellier Montpellier France; ^6^ Private office Hypnotherapist Montpellier France

**Keywords:** alternative medicine, breast cancer, chemotherapy

## Abstract

**Background:**

Acceptability and tolerance of chemotherapy on patients treated for breast cancer remain challenging. Complementary approaches such as hypnosis may have a favorable impact both at the time of announcing and during chemotherapy, due to the notorious anxiety, distress, and self‐perceived dysfunction. The objective of the study was that the patients complied with at least four self‐hypnosis sessions out of the six cycles of chemotherapy.

**Methods:**

This open, prospective longitudinal study assessed feasibility of compliance to self‐hypnosis during chemotherapy in an outpatients setting. Training sessions were given by a hypnotherapist. Throughout each cycle of chemotherapy, the patient had to use self‐hypnosis to better control her anxiety or any difficulties. Nurses could offer help to the patient. Chemotherapy‐associated side effects were evaluated through the NCI‐Common Toxicity Criteria for Adverse Events v 4.03; moreover, side effects as pain, nausea, vomiting, fatigue, and anxiety were also evaluated during chemotherapy using a visual analogic scale. Health‐related quality of life, emotional distress (anxiety and depression), and cancer‐related fatigue were assessed (at inclusion, end of chemotherapy and 3 months later) using the EORTC QLQ‐C30 and QLQ‐BR23, HADS and MFI‐20 questionnaires, respectively. The number of patients screened and actually included in the study was reported, as the reasons for refusal.

**Results:**

Thirty‐five patients were included with a median age of 55 years (35–78). All patients received a hypnosis training session. The overall compliance with self‐hypnosis was 68.6% (95% CI: 50.7%–83.2%), meaning that more than two thirds of patients performed at least four sessions of self‐hypnosis. According to NCI‐CTCAE, Grade 2 nausea and vomiting was observed in 45.7% and 22.9%, respectively, Grade 2 fatigue in 62.9%. Based on the HADS questionnaire, anxiety increased at the end of the chemotherapy and returned to the initial value 3 months later (*p* = .97) whereas depression significantly decrease 3 months after the end of chemotherapy with respect to the inclusion (*p* = .003). Role, emotional, and cognitive functioning were slightly affected throughout the treatment, in contrast to dyspnea or physical functioning.

**Conclusion:**

Our study showed that self‐hypnosis was feasible on patients newly diagnosed for breast cancer receiving chemotherapy.

## 1 INTRODUCTION

Patients treated for cancer by surgery, radiotherapy, and chemotherapy are vulnerable throughout the course of the disease. The period of time around the announcement of cancer and the onset of chemotherapy is likely to generate anxiety, distress, and self‐perceived dysfunction.^1^, ^2^ Quality of life scores have revealed impairments in role^3^ (i.e., personality, interaction between other persons, ability to daily, or leisure time activities), emotional or social function. Fatigue is very common in cancer care, described in more than 50% of the patients^4^; fatigue is often associated with other symptoms related to cancer disease or its treatment, as sleep troubles, anxiety, or cognitive impairment.^5^ In opposition to the technical care of breast cancer patients, these cognitive aspects may influence tolerance, side effects, acceptability of further treatment, and even reduce the risk of all‐causes of mortality.^6^ Cognitive behavioral therapy or more generally psychological approaches have demonstrated their favorable benefit on quality of life, anxiety, and depression in patients treated for breast cancer in a large meta‐analysis.^6^ More and more often, complementary alternative therapies are being used by patients as support for their conventional therapy.^7^ Moreover, in 2014 guidelines were published by the Society of Integrative Oncology to help physicians and patients to manage the treatment‐related symptoms of breast cancer, such as fatigue, chemotherapy‐induced nausea or vomiting, depression or global quality of life and physical alterations.^8^, ^9^, ^10^ By the way, the Continuous Update Project through their recommendations on limitations of drugs, smoking, and alcohol consumption or on dietary and physical activity involve the person as an actor in his lifestyle and consequently in cancer prevention.^10^ In considering the patient as an entity in terms of physical and psychological behavior, integrative medicine combine evidence‐based specific anticancer treatment or interventional practices, and alternative approaches, more dedicated to affects like distress or emotion.^11^, ^12^ Homeopathy, nutrition, psychological support, acupuncture, mind and body represent the most common used and well described elsewhere.^11^ A very large range of technics such as meditation, music therapy, yoga, relaxation, including hypnosis, also named complementary medicine, implying the patient directly, favoring self‐management strategies, have been evaluated in patients treated for breast cancer^13^ and have led to recommendations by the American Society of Clinical Oncology.^14^ Hypnosis is historically renowned for its numerous benefits.^15^ The American Society of Clinical Hypnosis describes hypnosis as “a state of inner absorption, concentration, and focused attention. It is like using a magnifying glass to focus the rays of the sun and make them more powerful. Similarly, when our minds are concentrated and focused, we are able to use them more powerfully. Because hypnosis allows people to use more of their potential, learning self‐hypnosis is the ultimate act of self‐control.”^14^ In France, in its report on Complementary Medicine, the Academic Society of Medicine considered hypnosis among other technics such as acupuncture, tai‐chi, and osteopathy in several clinical situations as bone pain, surgery or chemotherapy‐induced nausea or vomiting.^15^, ^16^ In general, despite of the lack sufficient number of patients or methodological limits, hypnosis showed a favorable impact on chemotherapy‐induced nausea or vomiting.^16^ The principle of clinical hypnosis is to change the state of consciousness, guided by a professional who attempts to shift the attention from one ongoing situation to another considered as more enjoyable. This first stage of relaxation is followed by a dissociative state under the hypnotherapist's control (hypnotic trance).^17^ This state of hypnotic trance allows patients to remain outside the current situation without distraction and focus on their own feelings, images, or thoughts.^18^ The goal of the technique, whatever the multiplicity of the situation (pain, anxiety, nausea‐vomiting, and so forth) remains to control these functional symptoms. The technic can be “Self‐Induced” by the patient, and used as a tool to control pain, anxiety, relaxation, or negative representations.^19^

### 1.1 Objectives and outcomes

Our main objective was to assess the feasibility of an intervention based on hypnosis as a complementary therapy in patients diagnosed with breast cancer and requiring adjuvant chemotherapy and radiotherapy. The primary endpoint was the proportion of patients who complied with the self‐hypnosis sessions during chemotherapy.

The secondary objectives were multiple, including the longitudinal assessment of the following patient‐reported outcomes: Quality of life using the general health‐related quality‐of‐life QLQ C30 and the QLQ‐BR23 module,^20^ anxiety and depression using the Hospital Anxiety and Depression Scale (HADS),^21^ and cancer‐related fatigue using the Multidimensional Fatigue Inventory (MFI‐20).^22^ These questionnaires were administered at inclusion into the study, at the end of chemotherapy and 3 months after the end of the chemotherapy.

The QLQ‐C30 questionnaire developed by the European Organization for Research and Treatment of Cancer (EORTC) is a validated cancer‐specific questionnaire based on 30 questions. It assesses five functional scales (physical, role, cognitive, social, and emotional), nine symptom scales (nausea and vomiting, pain, fatigue, dyspnea, sleeping disturbances, appetite loss, constipation, diarrhea), perceived financial difficulties and a global health status. The specific EORTC QLQ‐BR23 module is dedicated to breast cancer patients and includes 23 questions allowing to assess four functional scales (body image, sexual functioning, sexual enjoyment, future perspective) and four symptom scales (systemic side effects, arm symptoms, breast symptoms, being upset by the hair loss).

Other secondary objectives of our study were the evaluation of the chemotherapy‐induced side effects using the National Cancer Institute's Common Terminology Criteria for Adverse Events (NCI‐CTCAE v 4.03). In addition, visual analogic scales (VAS) were used to evaluate pain, nausea, vomiting, fatigue, and anxiety at each chemotherapy cycle.

Finally, the percentage of inclusion among eligible patients and the reason for their refusal was also evaluated.

### 1.2 Methods and design

This open, prospective, feasibility interventional single‐center study named HYPNOVAL was conducted at the Montpellier Cancer Institute in an outpatients' hospitalization setting. Patients were recruited from the active file of patients diagnosed with breast cancer. Eligibility criteria were the following: patients were all female aged ≥18 years who had never practiced hypnosis before, after primary surgery for breast cancer, and requiring adjuvant chemotherapy for at least 3 months followed by radiotherapy. All patients had to read and speak French and were exempt from any psychiatric disorders assessed on their medical history.

The trial, as well as details of chemotherapy and the expected side effects, was presented to the patient during the oncologic consultation. Once the patient had received oral and written information about the trial, a time of reflexion was offered in accordance with the regulatory requirements. All the patients gave their written consent. The protocol was approved by the French Ethical Committee “Sud Mediterranée III” on April 2015, EudraCT no 2014/A00745 42.

### 1.3 Study design

**FIGURE 1:**
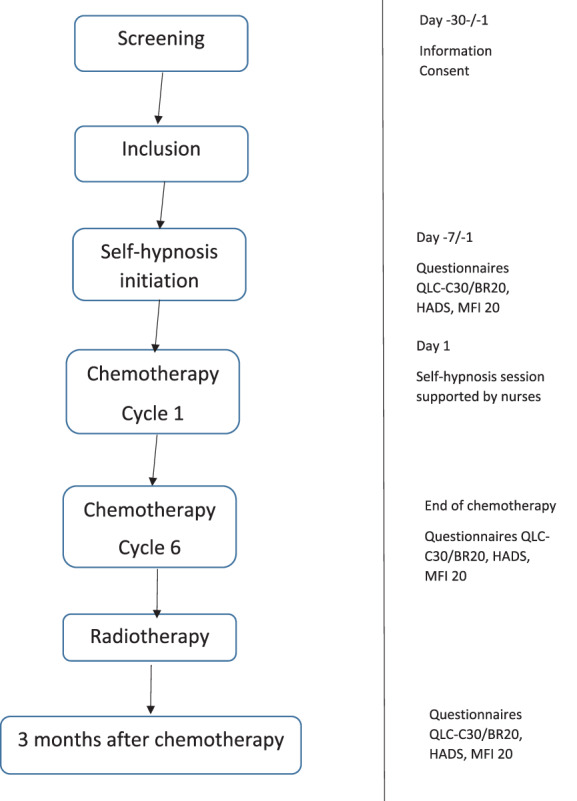
Flow chart

Once the patient consent to take part in the study, a consultation with a hypnotherapist was organized 1 week before the start of the chemotherapy. We had previously asked patients about their feelings towards hypnosis. The expected duration of the consultation was 45 min. The therapist assessed the patient's thoughts and feelings toward hypnosis, what therapeutic effects the patient expected from the hypnosis technique. Then, an explanation was given on how hypnosis works, that is, as a neuropsychological process induced by others (hetero‐hypnosis) or by oneself (self‐hypnosis), using one's own resources in memory and imagination. After this preliminary phase, the hypnosis session could begin (Figure 1).

### 1.4 Hypnosis technique

The therapist asked the patient to close her eyes in order to favor focus on their advices. The patient had to release herself from spatial and temporal surveillance, leading to a total body‐ and thought‐abandoning state; in a stretched‐out position, the patient could experience the full perception of this state. The patient and her therapist both ratified the situation. During this phase, no suggestions other than a reminder of what was initially exposed, that is that the patient had the possibility to reinitiate herself to the current state if needed (a reminder about the state of hypnosis). The patient was then considered as being capable of inducing self‐hypnosis.

### 1.5 Outpatient chemotherapy session

Ongoing treatments and vital signs were recorded. Every 3 weeks chemotherapy perfusion began at this point and the patient could use the self‐hypnosis technique to control her distress and anxiety; nurses trained in hypnosis techniques could help the patient go into a trance whenever required. The nurse who were able to help the patient received a specific training in a degree delivered at the university. When the patient asked the nurse to help her, she lessoned which difficulties the patient had and delivered advices to persevere in the approach. Then, the Clinical Research Assistant had a telephone call with the patient a few days after the cycle of chemotherapy; it was asked whether the patient has been able to perform spontaneously the self‐hypnosis session or whether she needed help from the nurse.

### 1.6 Toxicity/Tolerance

Chemotherapy‐induced side effects, concomitant treatment and the patient's ability to manage the self‐hypnosis session were evaluated by a telephone call 1 week after each cycle of chemotherapy, then at each day of hospitalization. Toxicity was assessed according to the NCI‐CTCAE, v 4.03. VASs were also used for pain, nausea, vomiting, fatigue, and anxiety.

### 1.7 Methodology and statistics

The primary endpoint was the proportion of patients who complied with the self‐hypnosis sessions during chemotherapy. Sample‐size calculation was based on this feasibility indicator. A patient was considered as compliant if she performed at least two thirds of the planned sessions (i.e., at least four out of six). In this context, by including 30 patients it would be possible to estimate a proportion of around 75% of patients complying with self‐hypnosis, with a 95% confidence interval (CI) (an interval of 0.3). Considering that 15% of patients were nonevaluable, 35 patients were to be included.

Statistical analysis: variables considered as categorical were described using frequencies and percentages, continuous variables using mean (standard deviation), median and range. VASs (ranged 0–10) used to assess some symptoms during chemotherapy were classified into three categories of severity [low (≤3/10), moderate (3–7), and severe or high (≥7/10)].

The EORTC QLQ‐C30 and QLQ‐BR23 questionnaires were analyzed following the EORTC guidelines.^20^ HADS scores assessing anxiety and depression were calculated and the scores were categorized according to the Zigmond classification (absence of disorder, suspected disorder, disorder). The MFI‐20 questionnaire was described according to the five dimensions subscales (General Fatigue, Physical Fatigue, Mental Fatigue, Reduced Activity, and Reduced Motivation).^22^

A nonparametric paired Wilcoxon test was used to compare quantitative variables (scores) between different times (i.e., end‐of‐chemotherapy vs. inclusion, 3 months after the end‐of‐chemotherapy vs. end‐of‐chemotherapy and 3 months after the end‐of‐chemotherapy vs. inclusion). The statistical significance level was set at *p* < .05. Data were analyzed using Stata version 13 (StataCorp LP, College Station, TX).

## 2 RESULTS

**FIGURE 2:**
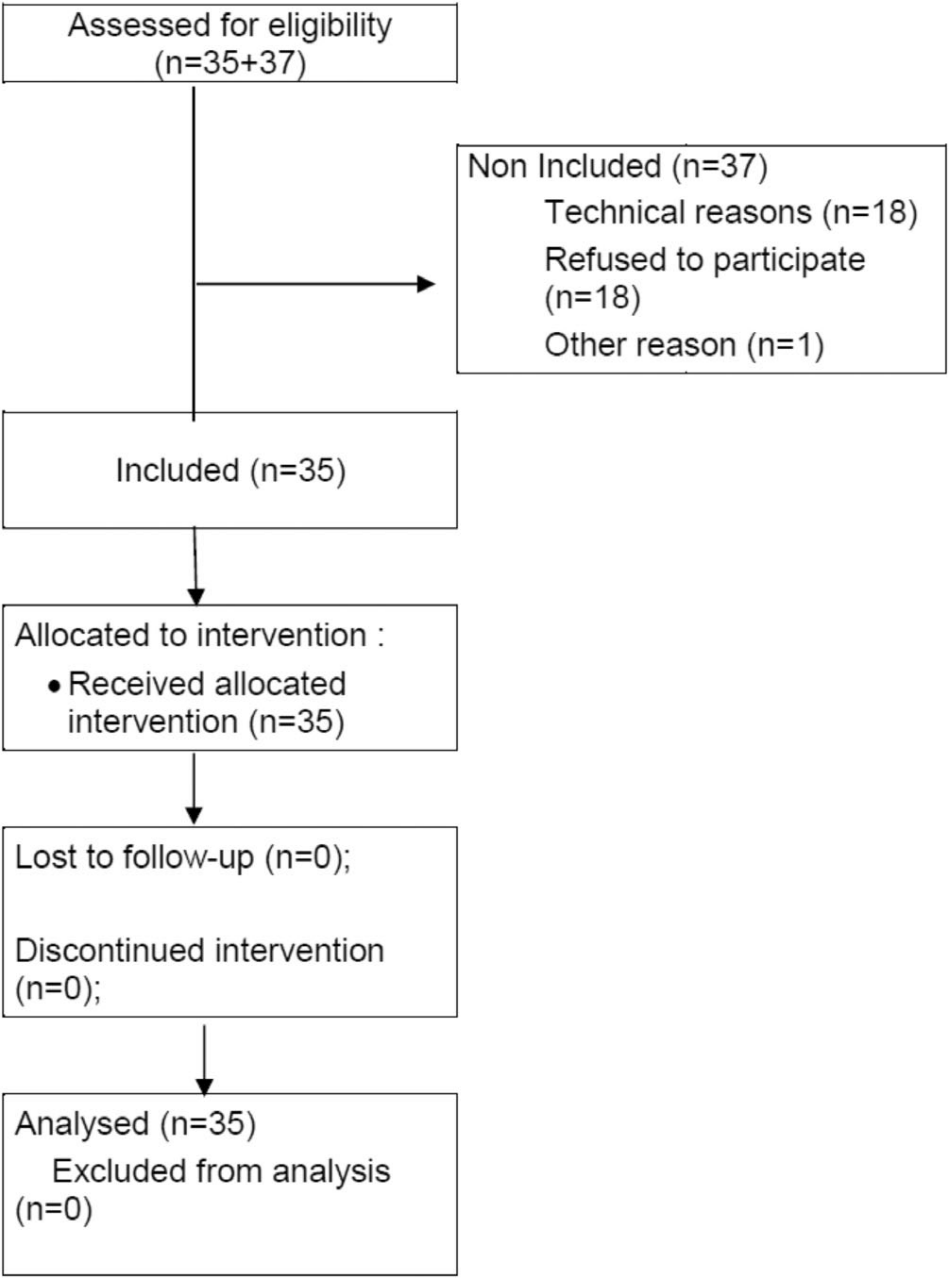
HYPNOVAL diagram

From July 2014 to June 2015, 72 patients were screened at the Montpellier Institute of Cancer and 35 patients were included, with a median age of 55 years (35–78). Nine (25.7%) underwent a mastectomy. Ninety‐seven percent of these patients received six cycles of chemotherapy. Among the patients who were not included, the main reason was technical difficulties in planning appointments for 18 of them; 19 other patients refused the inclusion for various reasons (Figure 2).

### 2.1 Hypnosis training session

The session was performed by a qualified physician before the patients began chemotherapy. All patients received a training lasting 45 min. To assess whether the patient had gone into a state of trance (median duration 30 min), the physician checked for closed eyelids, absence of body movement, hand catalepsy, and amimia. All the included patients experienced their own state of hypnosis.

### 2.2 Self‐hypnosis

At the first cycle of chemotherapy, 83% of patients performed a self‐hypnosis session but a slow decrease was observed throughout the treatment to finally reach 56% at the fifth cycle. At the last cycle, 66% of patients performed the technique. Fifty percent of patients asked the nurses for help at the first cycle versus 17% at the last cycle. The overall compliance with self‐hypnosis was 68.6% (95% CI: 50.7%–83.2%), meaning that more than two thirds of the patients performed at least four self‐hypnosis sessions among the six cycles of chemotherapy. The main reasons for not performing the self‐hypnosis session were as follows: patients felt they did not need it, presence of family/friends prevented it, pain due to recent deep intravenous implantation, desire to no longer practice hypnosis, less apprehension as the cycles progressed, help from friends or family.

The reasons why patients asked for help were the following: stress, anxiety, difficulty in practicing self‐hypnosis, need to check whether the patient was doing it correctly, no help offered during the last cycle. When the requested help was not obtained, the main raison was staff unavailability.

### 2.3 Secondary endpoints

#### 2.3.1 Chemotherapy‐induced toxicity/tolerance

According to the NCI‐CTCAE v4.03 criteria, 37.1% of patients presented Grade 2 toxicity, 37.1% had Grade 3 toxicity, and 22.9% had Grade 4. Grade 2 nausea and vomiting was observed in 45.7% and 22.9% of the patients, respectively. Grade 2 fatigue was observed in 62.9% of patients and increased to Grade 3 in only two patients (5.7%). There was no chemotherapy‐induced febrile neutropenia and no occurrence of severe side effects (Table 1).

**TABLE 1:** Evolution of depression/anxiety scores according to different time points in the study

HADS	Inclusion	End of CT	3 months after CT	End of CT vs. inclusion	3 months after end of CT vs. end of CT	3 months after end of CT vs. inclusion
	Median range	Median range	Median range	*p*‐value	*p*‐value	*p*‐value
Depression	8.0 [0; 16]	7.0 [0; 18]	6.0 [0; 16]	.025	.088	.003
Anxiety	3.0 [0; 13]	5.0 [0; 17]	3.0 [0; 11]	.217	.179	.967
Global score	11.0 [0; 27]	11.0 [2; 30]	9.0 [0; 27]	.533	.041	.03

According to the VAS, the proportion of patients with severe fatigue increased from 11.4% (Cycle1) to 30.3% (Cycle 6). Nausea and vomiting were generally of low grade and became moderate during Cycles 3–4 (12.5% and 21.9%, respectively). They were considered as severe only in 3% of all chemotherapy patients. Four patients (11.4%) complained of moderate pain at Cycle 1 and 17 patients (53%) at Cycle 4. Severe pain was observed in 12.5% of patients. Nevertheless, high well‐being declined from 65.7% (Cycle 1) to 25% (Cycle 4), reaching the initial values at Cycle 6 (69.7%).

#### 2.3.2 Hospital Anxiety and Depression Scale

All the patients filled in the questionnaires upon inclusion, at the end of the chemotherapy and 3 months later. The global scores were 11 [0; 27], 11[2; 30], and 9 [0; 27], respectively. The depression scale varied from 8 [0; 16] to 7 [0; 18] and 6 [0; 16]. A significant reduction in the global score and the depression scale was observed 3 months after the end of chemotherapy with respect to the inclusion (*p* = .03 and *p* = .003, respectively). Finally, anxiety had increased at the end of the chemotherapy and returned to the initial value 3 months later (*p* = .97), with scores varying from 3 [0; 13], 5 [0; 17] to 3 [0; 11]. Details of depression and anxiety according to severity (presence vs. absence) are shown in Table 2.

**TABLE 2:** Multidimensional Fatigue Inventory

MFI	Inclusion	End of CT	3 months after end of CT	End of CT vs. inclusion	3 months after end of CT vs. end of CT	3 months after end of CT vs. inclusion
	Median range	Median range	Median range	*p*‐value	*p*‐value	*p*‐value
General fatigue	23.0 [9; 37]	32.0 [9; 45]	23.0 [9; 44]	.0001	.002	.111
Mental fatigue	12.0 [6; 24]	15.0 [6; 25]	13.0 [6; 26]	.018	.025	.388
Reduced activity	7.0 [3; 13]	10.0 [3; 15]	6.0 [3; 14]	.005	.008	.361
Reduced motivation	4.0 [2; 9]	5.0 [2; 9]	4.0 [2; 9]	.614	.359	.708

#### 2.3.3 Multidimensional Fatigue Inventory

With the exception of the reduce motivation dimension, all fatigue dimensions (general fatigue, mental fatigue, physical fatigue, and reduced active) increased significantly from baseline to the end of chemotherapy (*p* < .05). Three months after the end of chemotherapy the scores reached the initial values excepted for the general fatigue dimension (median score varied from 10 [4; 16], 14 [4; 20], and 12[4; 20] at inclusion, end of chemotherapy and 3 months after the end of chemotherapy) (Table 3).

**TABLE 3:** Multidimensional Fatigue Inventory (MFI‐20)

MFI‐20	Inclusion	End of CT	3 months after end of CT	End of CT vs. inclusion	3 months after end of CT vs. end of CT	3 months after end of CT vs. inclusion
	Median range	Median range	Median range	*p*‐value	*p*‐value	*p*‐value
General fatigue	10 [4; 16]	14 [4; 20]	12 [4; 20]	<.0001	.0046	.0289
Mental fatigue	8 [4; 16]	11 [4; 18]	8 [4; 19]	.0282	.0344	.5682
Physical fatigue	10 [4; 16]	14 [4; 20]	10 [4; 20]	.0021	.0044	.5279
Reduced activity	9 [4; 18]	14 [4; 20]	9 [4; 18]	.0014	.0056	.2407
Reduced motivation	8 [4; 17]	10 [4; 16]	9 [4; 16]	.5303	.0758	.9607

#### 2.3.4 Quality of life

According to the EORTC QLQ‐C30 questionnaire (Table 4) we observed a significant deterioration of physical and social functioning at the end of chemotherapy compared with the baseline (median scores varies from 66.7 to 50.0 (*p* = .0001) and from 83.3 to 66.7 (*p* = .034), respectively). At the same time, symptoms as fatigue and dyspnea significantly increase (median scores varies from 33.3 to 66.7 and from 0.0 to 33.3, respectively; *p* = .0007).

**TABLE 4:** Evolution of QLQ‐C30 B23 module scores

Dimension QLQ‐BR23	Inclusion	END of CT	3 months after End CT			
	Median range	Median range	Median range	End of CT vs. Inclusion *p*‐value	3 months after CT vs. end of CT *p*‐value	3 months after end of CT vs. inclusion *p*‐value
Body image				.0005	.103	.044
	75.0 [0; 100]	50.0 [0; 100]	66.7 [0; 100]			
Sexual functioning				.7142	.042	.010
	16.7 [0; 66.67]	16.7 [0; 66.67]	25.0 [0; 66.67]			
Sexual enjoyment				.298	.342	.257
	33.3 [0; 100]	66.7 [0; 100]	66.7 [0; 100]			
Future prospects				.023	.666	.005
	66.7 [0; 100]	66.7 [0; 100]	66.7 [0; 100]			
Systemic therapy side effects				.00	.0000	.004
	9.5 [0; 66.67]	43.3 [14.29; 80.95]	19.0 [0; 57.14]			
Breast symptoms				.000	.000	.28
	25.0 [0; 83.33]	8.3 [0; 41.67]	25.0 [0; 83.33]			
Arm symptoms				.0003	.112	.015
	22.2 [0; 66.67]	11.1 [0; 55.56]	11.1 [0; 66.67]			
Upset by hair loss				.317	.578	…
	66.7 [33.33; 100]	33.3 [0; 100]	33.3 [0; 100]			

For the vast majority of the domains explored by the QLQ C30 questionnaire, 3 months after the end of chemotherapy, the scores returned to those recorded at baseline (Table 4). For example role, emotional and cognitive functioning were marginally affected throughout the treatment, in contrast to dyspnea or physical functioning, which were impacted at the end of treatment. Considering BR23 module‐captured items specific of breast cancer; the body image score decreased significantly throughout chemotherapy. The sexual functioning score was considered as stable, in contrast with the systemic therapy side effects score, which increased significantly throughout the treatment (Table 5).

**TABLE 5:** Evolution of QLQ‐C30 B23 module scores

Dimension QLQ‐BR23	Inclusion	END of CT	3 months after End CT			
	Median range	Median range	Median range	End of CT vs. Inclusion *p*‐value	3 months after CT vs. end of CT *p*‐value	3 months after end of CT vs. inclusion *p*‐value
Body image				.0005	.103	.044
	75.0 [0; 100]	50.0 [0; 100]	66.7 [0; 100]			
Sexual functioning				.7142	.042	.010
	16.7 [0; 66.67]	16.7 [0; 66.67]	25.0 [0; 66.67]			
Sexual enjoyment				.298	.342	.257
	33.3 [0; 100]	66.7 [0; 100]	66.7 [0; 100]			
Future prospects				.023	.666	.005
	66.7 [0; 100]	66.7 [0; 100]	66.7 [0; 100]			
Systemic therapy side effects				.00	.0000	.004
	9.5 [0; 66.67]	43.3 [14.29; 80.95]	19.0 [0; 57.14]			
Breast symptoms				.000	.000	.28
	25.0 [0; 83.33]	8.3 [0; 41.67]	25.0 [0; 83.33]			
Arm symptoms				.0003	.112	.015
	22.2 [0; 66.67]	11.1 [0; 55.56]	11.1 [0; 66.67]			
Upset by hair loss				.317	.578	…
	66.7 [33.33; 100]	33.3 [0; 100]	33.3 [0; 100]			

## 3 DISCUSSION

The aim of the HYPNOVAL study was to demonstrate the feasibility of self‐hypnosis in patients treated for the first time by adjuvant chemotherapy for breast cancer. We showed that the overall compliance with self‐hypnosis was 68.6% (95% CI: 50.7%–83.2%), meaning that more than two thirds of the patients performed at least four self‐hypnosis sessions among the six cycles of chemotherapy. According to the 95% CI estimated in our sample, we can expect that the compliance with self‐hypnosis in a general population of breast cancer patients upon diagnosis of their disease would vary between 51% and 83% (which comprises the hypothesized proportion of 75%). Tolerance of chemotherapy remains challenging and, despite all the information given through the media and by the oncologic teams, its representation in the patient's imagination may influence the patient's behavior before and during treatment.^8^, ^23^ The role of complementary medicine in this area has been defined by several studies and is the subject of guidelines published in the Journal of Clinical Oncology in 2018.^14^ As combination‐based approaches and the interactions of numerous permutations of complementary medicines and conventional treatments had not been formally investigated, new studies were required to better assess their role, in this case, self‐hypnosis. Moreover, introducing these techniques in the outpatient chemotherapy setting requires extra efforts and coordination from the teams and involves the intervention of qualified staff, more specifically in our study, a dedicated physician trained in hypnosis. More data on the human time spent and the cost incurred are required in view of the expected benefits.

HYPNOVAL study showed that recruitment was possible in a selected population of newly diagnosed early breast cancer patients. Out of 72 patients screened, 35 (48.6%) participated in the study. The reasons for not taking part in the study were various: in half the cases, we encountered technical difficulties in planning the exams and giving the self‐hypnosis training. Some patients refused due to the apparent complexity of the protocol, feeling that it would involve additional constraints like coming back to the hospital, especially for those who lived far away from the center. Some patients also refused because they did not believe in the efficacy of hypnosis.

For those patients entering the study, all of them complied with the hypnosis training session, in reaching hand catalepsy, amimia and state of trance.

Our study showed that patients who were unfamiliar with hypnosis were able to learn the techniques and use them throughout the chemotherapy cycles. That way, 68.6% (more than two thirds) of patients performed at least four sessions of self‐hypnosis among the six chemotherapy cycles. However, we are aware that one limitation of our method is the time dedicated to hypnosis training; in order to make that hypnosis can be considered as appropriated by the patient, a more intense training will be useful, as it was planned by other researcher.^19^

However, the percentage of practicing patients decreased from the beginning (83%) to the end (66%) of chemotherapy. At the first cycle, 50% of patients asked the nurses for help. Disturbances like the presence of family, noise, difficulty to self‐practice, or the need for someone to check whether the patient had anxiety or pain may have contributed to preventing self‐hypnosis. In a way, this appeared paradoxical as the expected efficacy of hypnosis against anxiety or pain is well‐known. The availability of staff to help the patient with self‐hypnosis appeared to be a challenge because not all the outpatient facility's nurses were trained in hypnosis techniques or had enough free time to devote to hypnosis. It was interesting that a quiet environment was requested to reach the introspection necessary for self‐hypnosis.

Out of the 35 patients included, QLQ‐C30, MFI, HADS questionnaires were completed quite regularly, even at 3 months after the end of radiotherapy. Due to the noncomparative, feasibility nature of our study, its purpose was not to assess the impact of self‐hypnosis on tolerance and quality of life or anxiety‐depression in women receiving chemotherapy for breast cancer. After all, the scores recorded for the different domains went back to the initial level at 3 months. For example, anxiety was slightly greater at the end of the chemotherapy and decreased progressively.^24^ The level of depression decreased regularly.^25^

In conclusion, HYPNOVAL study has shown that including self‐hypnosis throughout chemotherapy in naive breast cancer patients was feasible, with the need to adjust internal organization. The impact of chemotherapy on anxiety, depression, fatigue and, in general, on the quality of life index warrants further investigations by means of a larger comparative study devoted to assess the efficacy of self‐hypnosis.

## AUTHOR CONTRIBUTIONS

**Michel Fabbro:** Conceptualization (lead); formal analysis (lead); funding acquisition (lead); investigation (lead); supervision (equal); writing – original draft (lead). **William Jacot:** Investigation (equal); writing – review and editing (equal). **Marta Jarlier:** Formal analysis (lead); methodology (equal); writing – review and editing (equal). **Séverine Guiu:** Investigation (equal); writing – review and editing (equal). **Véronique D'Hondt:** Investigation (equal); writing – review and editing (equal). **Stéphane Pouderoux:** Investigation (equal); writing – review and editing (equal). **Patrice Champoiral:** Writing – review and editing (equal). **Chloé Janiszewski:** Project administration (equal); writing – review and editing (equal). **Isabelle Nicklès:** Conceptualization (equal); investigation (equal); writing – review and editing (equal).

## CONFLICT OF INTEREST

All the authors declare having no conflict of interest.

## ETHICS STATEMENT

The HYPNOVAL protocol had been approved by the Sud Mediterranée III French Ethical Committee (April 2015) and registered on ID‐RCB no 2014‐A00745‐42 and at Clinicaltrials.gov (NCT03250130).

## Data Availability

The data that supports the finding of this study are available from the corresponding author on reasonable request.
